# Excimer Laser Induced Spatially Resolved Formation and Implantation of Plasmonic Particles in Glass

**DOI:** 10.3390/nano8121035

**Published:** 2018-12-12

**Authors:** Maximilian Heinz, Jörg Meinertz, Manfred Dubiel, Jürgen Ihlemann

**Affiliations:** 1 Institute of Physics, Martin Luther University Halle-Wittenberg, Von-Danckelmann-Platz 3, D-06120 Halle (Saale), Germany; maximilian.heinz@physik.uni-halle.de (M.H.); manfred.dubiel@physik.uni-halle.de (M.D.); 2 Laser-Laboratorium Göttingen e.V. Hans-Adolf-Krebs-Weg 1, D-37077 Göttingen, Germany; joerg.meinertz@llg-ev.de

**Keywords:** plasmonic nanoparticles, laser implantation, interference pattern

## Abstract

Metallic nanoparticles are important building blocks for plasmonic applications. The spatially defined arrangement of these nanoparticles in a stable glass matrix is obtained here by nanosecond excimer laser irradiation at 193 nm. Two approaches are addressed: (1) Laser induced formation of particles from a dopant material pre-incorporated in the glass, (2) Particle formation and implantation by irradiation of material pre-coated on top of the glass. Silver nanoparticles are formed inside Ag^+^ doped glass (method 1). Gold nanoparticles are implanted by irradiation of gold coated glass (method 2). In the latter case, with a few laser pulses the original gold film disintegrates into particles which are then embedded in the softened glass matrix. A micron sized spatial resolution (periodic arrangements with 2 µm period) is obtained in both cases by irradiating the samples with an interference beam pattern generated by a phase mask. The plasmonic absorption of the nanoparticles leads to a contrast of the optical density between irradiated and non-irradiated lines of up to 0.6.

## 1. Introduction

The generation and controlled arrangement of metallic nanoparticles is very important for the fabrication of plasmonic elements. Silver and gold nanoparticles in glasses are attracting particular attention since the wavelength of their surface plasmon resonance (SPR) is in the visible spectral range, making such materials promising candidates for applications in optoelectronics and nanoplasmonics [1,2]. Typically, silver nanoparticles in sodium silicate glasses are prepared by a Ag^+^↔Na^+^ ion exchange process or ion implantation and subsequent thermal treatment [3,4,5]. However, this method does not allow the formation of particles localized in a certain way. For spatially selective particle formation, local heating of ion exchanged sodium silicate glasses by laser irradiation can be utilized [6,7,8]. Short and ultrashort pulsed lasers have been used to produce a variety of nanostructures for several applications. Besides creating hole or line patterns in metal films [9,10,11], they can also be utilized for localized particle formation on or in glass. In the case of Ag particle formation in ion exchanged glass, this process can be accomplished by multipulse excimer laser irradiation (ArF, 193 nm) at fluences below the ablation threshold [12], at 248 nm [13], or at 355 nm [14]. Also combined thermal and laser processes are applied [15,16,17]. Special local arrangements of Ag particles have been obtained by femtosecond laser focusing [18], by the so called laser induced periodic surface structuring process (LIPSS) [19], or interference concepts [20,21]. However, shrinking [22] and dissolution [23] of nanoparticles by laser irradiation have also been observed.

As glass cannot be doped with a sufficient concentration of gold in a similar way, for the near-surface formation of gold particles in glass, a different method is applied: the glass surface is coated with a thin gold film and subsequently irradiated with an excimer laser. Moderate laser fluences lead to dewetting of the gold film and particle formation. This has been shown besides for gold [24,25,26], and also for silver [27], nickel [28], and molybdenum [29] films. Shape, size, and coalescence behavior of the particles can be controlled by the applied laser parameters [30,31,32,33]. Structured irradiation leads to the arrangement of particles in corresponding areas [34,35,36,37] or localized bump or spike formation [38,39]. Methods for patterning of thin metal films by controlled dewetting have been reviewed by Ruffino et al. [40]. At sufficiently high fluence, the particles are in part or completely embedded or implanted into the glass [41]. A similar result is obtained when heating the coated glass by CO_2_-laser irradiation [42] or furnace annealing [43]. Using an ArF excimer laser at a fluence below the threshold of ablation, gold nanoparticles are formed and partially implanted into the glass as can be seen from the colored appearance of the irradiated areas and the plasmon resonance peak in the absorption spectra [44]. After wiping off the loosely sticking particles, the remaining effect is only due to the implanted particles. Here also, spatially defined irradiation leads to localized formation and implantation of particles.

In this paper we demonstrate that patterned irradiation of Ag^+^-doped glass using phase mask projection allows for the spatially resolved formation of Ag particles in the glass on large areas with µm-resolution. Using the same irradiation scheme, even the implantation of gold particles into the glass with the same—up to now unrivaled—spatial resolution is achieved.

## 2. Materials and Methods

For silver incorporation and Ag nanoparticle formation glass slides of 1 mm thickness of commercial soda-lime float glass containing (in wt%) 72% SiO_2_, 13.3% Na_2_O, 9% CaO, and 4% MgO as main components are used. After cleaning the slides with acetone, the silver ions are introduced into the glass network by an isothermal Ag-Na ion exchange in a salt melt with 5 wt% AgNO_3_ in NaNO_3_ at 330 °C for 20 min.

For the gold implantation experiments, glass samples of the same type have been cleaned with acetone and then sputter coated with gold (thickness 70 nm) on the tin-bath side of the float glass (Sputter coater EMITECH K550, Ashford, UK; current: 20 mA, Ar pressure: 10^−1^ mbar). 

**Figure 1:**
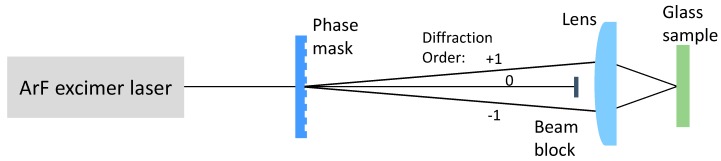
Laser irradiation set-up.

Spatially resolved irradiation is performed with an ArF excimer laser (Lambda Physik LPX315, Göttingen, Germany; wavelength 193 nm, pulse duration 20 ns) in combination with a mask projection setup (Figure 1). The choice of 193 nm for the laser wavelength is based on the fact that not only the metal, but also the glass matrix is strongly absorbing in this spectral range assisting the temporally and spatially controlled heating. A binary fused silica phase mask is illuminated by the laser beam and imaged on the sample by a UV-transparent achromatic lens (focal length 100 mm) with a demagnification factor of m = 8 (Ag-experiments) or m = 10 (Au-experiments). The phase mask consists of a relief line grating and is designed in a way that most of the transmitted light is diffracted in the ± first orders and the zero order intensity is minimized [45]. The residual zero order and the higher orders are blocked, so that only the ± first orders contribute to the image formation leading to a sin^2^ intensity pattern with a period d = D/(m × 2), if D is the period of the phase mask. Phase masks with D = 40 µm and D = 20 µm have been applied in the case of Ag-doped glass leading to periods on the sample of d = 2.5 µm and d = 1.25 µm, respectively. In the case of Au-coated glass, phase masks with D = 60 µm and D = 40 µm have been applied leading to periods on the sample of d = 3 µm and d = 2 µm, respectively. The image field size on the sample amounts to 200 µm × 200 µm. The laser pulse repetition rate is 10 Hz in the case of Ag-doped glass and 1 Hz in the case of Au-coated glass. The moderately higher rate of 10 Hz is chosen to speed up the multipulse process without leading to unwanted cumulative heating as expected for very high repetition rates. 

The resulting modifications are investigated with a UV-Vis-NIR microscope photometer MPM 100 (Carl Zeiss, Jena, Germany). The minimum width of the measurement area for recording transmission spectra is 1 µm. In addition, scanning electron microscopy (SEM, Zeiss EVO MA10, Oberkochen, Germany) and scanning transmission electron microscopy (STEM, FEI Titan3 80-300, Hillsboro, OR, USA, acceleration voltage of 200 kV) are applied.

## 3. Results

### 3.1. Formation of Ag-Nanoparticles

**Figure 2:**
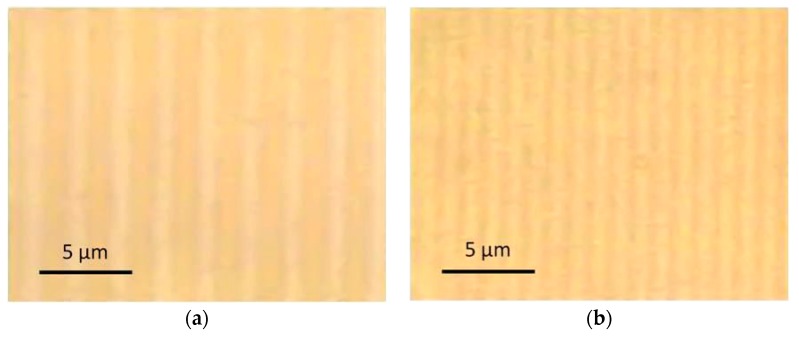
Microscope images (transmitted light) of line patterns obtained by structured irradiation of Ag-doped glass using a 40 μm-phase mask (**a**) and a 20 μm-phase mask (**b**). Parameters: 100 mJ/cm^2^ peak fluence, 1000 pulses.

**Figure 3:**
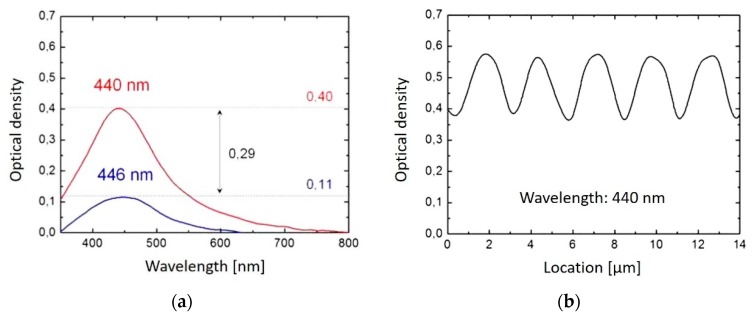
Spectra recorded with the UV-Vis-NIR microscope photometer at the line pattern obtained with the 40 µm-phase mask. (The absorption of the basic glass without Ag is subtracted.) (**a**) measurement at a dark line (red) and between two dark lines (blue) of a pattern made with 500 pulses. (**b**) absorption at 440 nm as a function of the position on a line pattern made with 1000 pulses.

Figure 2 displays microscopic views of the modified areas. The periodic patterns are clearly visible. The periods are in agreement with the above calculated values. Figure 3a shows absorption spectra recorded in the dark and in the bright area of the pattern, respectively. The plasmon resonance peak of Ag at about 440 nm indicating the formation of Ag nanoparticles is clearly visible, however it is much stronger in the dark area compared to the bright area. Figure 3b displays the course of the absorption at the peak wavelength of 440 nm when scanning the sensor slit across the pattern.

The size of the silver particles is concluded from the measurements performed under flat field irradiation with similar parameters [12]. Small particles of about 1–3 nm and agglomerations of these particles with 5–15 nm diameter have been found.

### 3.2. Formation of Au-Nanoparticles

**Figure 4:**
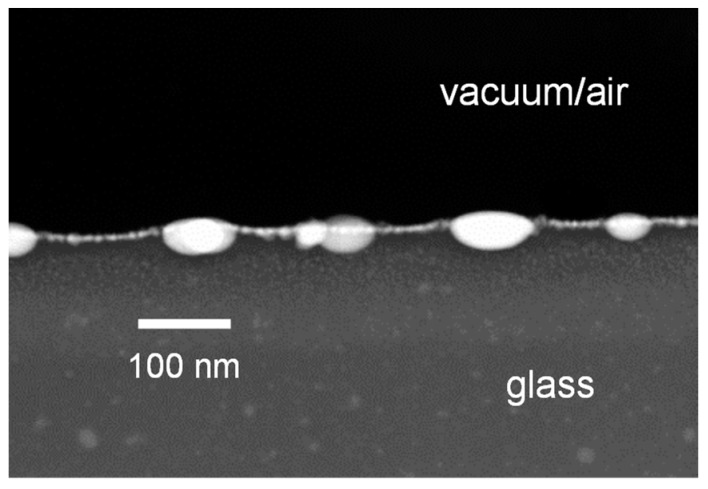
TEM image of a cross section through a gold coated glass surface after flat field (non-structured) irradiation with 10 laser pulses.

Figure 4 displays a TEM image of a cross section through a gold coated glass surface after flat field (non-structured) irradiation with 10 laser pulses. Large and small gold particles are positioned below the glass surface. 

**Figure 5:**
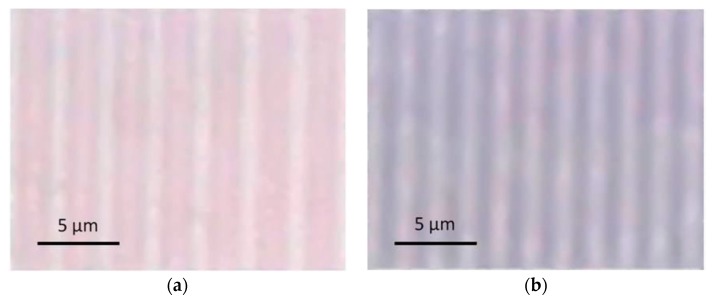
Microscope images (transmitted light) of line patterns obtained by structured irradiation of Au-coated glass (bath side) using a 60 μm-phase mask and a fluence of 260 mJ/cm^2^, 50 pulses (**a**), and a 40 μm-phase mask and a fluence of 400 mJ/cm^2^, 50 pulses (**b**). Both images show only the effect of implanted material after cleaning the surface with acetone.

**Figure 6:**
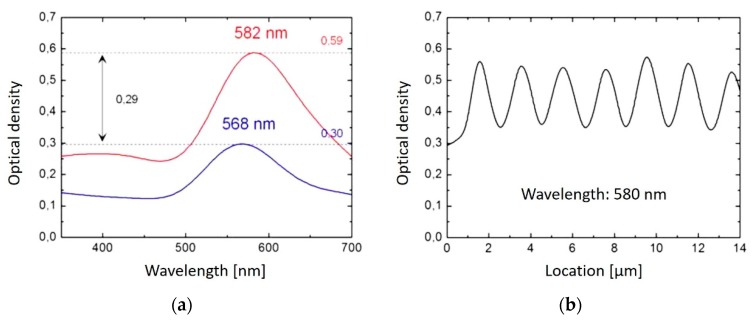
Spectra recorded with the UV-Vis-NIR microscope photometer at the line pattern obtained with the 40 µm-phase mask at 400 mJ/cm^2^, 50 pulses. (The absorption of the basic glass without Au is subtracted.) (**a**) measurement at a dark line (red) and between two dark lines (blue). (**b**) absorption at 580 nm as a function of the position.

Figure 5 displays microscopic views of the modified areas made by structured irradiation. Also here, the periodic patterns are clearly visible and the periods are in agreement with the above calculated values. Figure 6a shows absorption spectra recorded in the dark and in the bright area of the pattern, respectively. The plasmon resonance peak of Au at about 580 nm is clearly visible, however it is much stronger in the dark area compared to the bright area. Figure 6b displays the course of the absorption at the peak wavelength of 580 nm when scanning the sensor slit across the pattern.

The size of the spherical particles is assumed to be around 10 nm after 5 pulses and increasing to about 20 nm after 50 pulses according to the measurements performed under flat field irradiation with similar parameters [44]. However, some large, oblate particles (50 nm) are also present as can be seen from Figure 4. 

**Figure 7:**
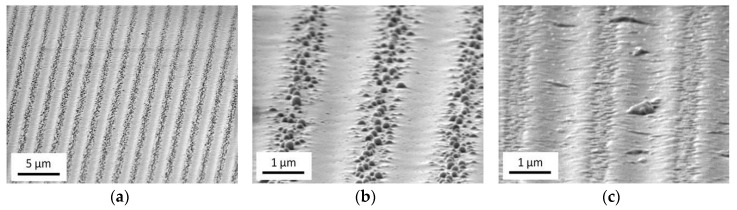
SEM-images (tilt angle 80°) of a line pattern made with the 40 µm-phase mask and a fluence of 400 mJ/cm^2^ (50 pulses) on a gold coated glass; Surface before (**a**,**b**) and after (**c**) cleaning with acetone.

Figure 7 displays the surface of a gold-coated glass after irradiation with a line intensity pattern with 2 µm period recorded by scanning electron microscopy (SEM). From the images (a) and (b) taken before cleaning the surface with acetone, rows of rather big particles can be found with a period corresponding to the irradiation pattern. After wiping off these particles, a pattern with a slight height variation can be seen (c). The implanted particles are still there (cf. Figure 5), but cannot been detected by the relief contrast operation mode of the SEM. The shallow grooves between the (removed) particle rows represent the lines of high laser intensity. It is obvious that the optical surface quality has been impaired by this process to some extent.

**Figure 8:**
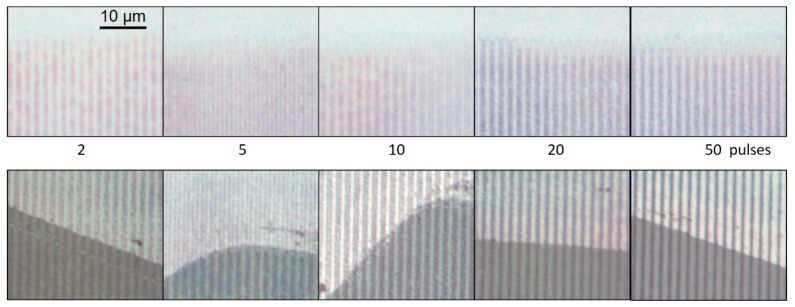
Microscope images (transmitted light) of implanted line patterns after irradiation with different numbers of pulses. Upper row: Outer area of the spots showing the line ending. Lower row: Boundary between cleaned (**top**) and as irradiated (**bottom**) area.

To locate the position of the implanted nanoparticles with respect to the irradiation pattern, the outer area of the line pattern (at the end of the lines) has been recorded by transmission light microscopy (Figure 8, top row). At low pulse number (2) the particle lines (dark) coincide with the high intensity lines. Going to higher pulse numbers (5–10), these dark lines split into double lines with a bright center, which merge into single lines again at even higher pulse numbers (20–50) and surround the now bright high intensity positions, as can be especially seen at the end of the lines.

Figure 8 (bottom row) displays images of the samples, where in the upper part the loosely adhering gold material is removed, and in the lower part of the image it is still present. One can see that at low pulse numbers (2) the bright lines (high transmission) in the two regions do not coincide; the implanted material (dark, upper part) is at the high intensity position, whereas the loose material (dark, lower part) is mainly transferred to the low intensity position. At higher pulse numbers also the implanted material moves to the low intensity positions.

## 4. Discussion

### 4.1. Formation of Ag-Nanoparticles

The obtained absorption spectra (Figure 3) are comparable to those recorded in the case of large area homogeneous irradiation of Ag-doped glass [12]. The plasmon absorption peaks at 446 nm (low fluence positions) and 440 nm (high fluence positions) obtained after 500 pulses with structured irradiation are similar to those from 450 nm (after 500 pulses) to 435 nm (after 5000 pulses) for large area illumination. This indicates that Ag-particles with radius < 3 nm and agglomerations > 5 nm have been formed [12]. It is assumed that this particle formation takes place in a locally softened or even liquefied glass matrix, so that the diffusion of the silver ions is fast enough to form particles during the effective laser heating period of only µs to ms duration when applying a few 100 to 5000 pulses [6,44].

Even at the low intensity positions a considerable plasmon absorption indicating particle formation is observed. This has several reasons: (1) The spatial irradiation profile is sinusoidal and not rectangular, (2) The finite size of the measurement slit integrates always over the laterally extended area, (3) Thermal diffusion will lead to some heat spreading into the regions of the intensity minima. However, the existence of a strongly modulated periodic pattern after a large number of laser pulses supports the perception of pulsed heating and subsequent cooling of the sample prior to the following pulse, as cumulative heating would certainly equalize the particle distribution and erase the contrast. 

The contrast C of the line patterns generated by the formed particles is defined by C = (OD_max_ − OD_min_)/(OD_max_ + OD_min_), where OD_max_ is the optical density OD = log_10_ (I_0_/I) at the particle rich positions and OD_min_ is the optical density in between these positions (I_0_ incoming intensity, I transmitted intensity). In the case of formed silver particles, the contrast amounts to C_Ag_ = (0.40 − 0.11)/(0.40 + 0.11) = 0.57 (cf. Figure 3). However, due to the finite slit width in the measurements, the measured contrast is diminished with respect to the real contrast. As the slit width itself cannot be measured with high accuracy, a real contrast of 1.1–1.5 times the measured contrast is estimated assuming a slit width of 0.5 to 1 µm. 

### 4.2. Implantation of Au-Nanoparticles

**Figure 9:**
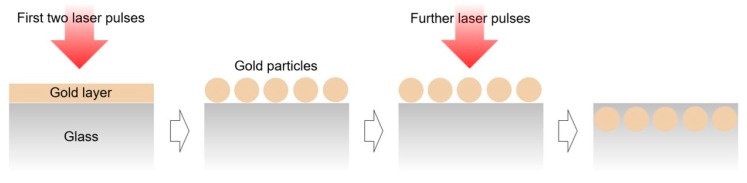
Scheme of particle formation and implantation. The first pulses lead to nanoparticle formation; further pulses cause implantation of the particles into the glass matrix.

The implantation of gold particles previously investigated for the case of homogeneous large area laser irradiation [44] is assumed to proceed according to the scheme shown in Figure 9. The first two laser pulses lead to disintegration of the smooth gold film and particle formation. Further pulses lead to increase of the temperature of the glass surface and melting processes. As a result, the gold particles are embedded in the softened glass matrix.

Whereas the spatially resolved formation of Ag-particles in the glass proceeds in an analogous manner compared to large area irradiation, in the case of the implantation, the lateral movement of material cannot be neglected. As seen in Figure 8, the implantation starts at the high intensity positions, but during further pulses the particles seem to be somehow transferred in direction to the non-irradiated area. A similar behavior of lateral displacement is observed when going from low to high fluences [46]. There it is explained by reablation of particles at high irradiation dose and redistribution or displacement of particles to the side areas. The movement of material in a temperature gradient can take place as motion of nanoparticles as a whole [21] and/or by thermodiffusion of atomic (dissolved) material [47]. Nevertheless, obviously this does not prevent a high spatial resolution of particle implantation. However, the process window for strongly localized implantation without reablation or lateral spreading seems to be rather narrow. Though data concerning the mobility of the metal in the glass matrix are available [48,49], due to the complex and dynamic temperature distribution, a detailed description of the involved processes would be rather speculative and go beyond the scope of this work. 

Regarding the contrast C = (OD_max_ − OD_min_)/(OD_max_ + OD_min_) of the line patterns generated by the implanted particles, for the structure period of 2 µm C_Au_ = (0.59 − 0.30)/(0.59 + 0.3) = 0.33 is obtained (cf. Figure 6). Again, the actual value will be 1.1–1.5 times higher due to the finite slit width in the measurement. Of course, the contrast will diminish when going to smaller structure periods. It does not depend only on the contrast of the optical irradiation pattern (sinusoidal in the case of two beam interference), but also on the lateral redistribution of material by induced temperature and viscosity gradients of the glass and effects like thermodiffusion, reablation, and redeposition during the course of several laser pulses. That these effects play a more dominant role than in the case of Ag-doped glass seems understandable, as the peak fluence of 400 mJ/cm^2^ required to obtain high contrast is significantly higher than that used for Ag-particle formation (100 mJ/cm^2^). It is already near the ablation threshold of 430 mJ/cm^2^ of the basic soda lime glass [50].

## 5. Conclusions

Periodic arrangements of plasmonic nanoparticles in glass with periods down to 1–2 µm have been obtained by structured excimer laser irradiation of Ag-doped glass and Au-coated glass, respectively. The contrast C of the plasmonic absorption of implanted gold between irradiated and non-irradiated lines of 1 µm width (2 µm period) amounts to C > 0.33. In the case of generated silver particles the contrast between irradiated and non-irradiated lines of 1.25 µm width (2.5 µm period) is even higher (C > 0.57). In order to obtain sub-micron patterns, the optical resolution of the set-up can easily be enhanced. However, thermal and material diffusion may limit the achievable contrast.
